# Natural Products in Diabetes Mellitus: 2nd Edition

**DOI:** 10.3390/ph18111666

**Published:** 2025-11-04

**Authors:** Ana A. Feregrino-Pérez

**Affiliations:** Posgrado en Ingeniería en Biosistemas, Facultad de Ingeniería, Universidad Autónoma de Querétaro, Carretera a Chichimequillas s/n km1, El Marques C.P. 76265, Mexico; feregrino.angge@hotmail.com

## 1. Introduction

Diabetes mellitus (DM) remains one of the most prevalent and challenging chronic metabolic disorders worldwide, characterized by persistent hyperglycemia due to impaired insulin secretion, insulin resistance, or both. According to recent global health reports, approximately 11.1% of adults aged 20–79 currently live with diabetes, with type 2 diabetes mellitus (T2DM) representing more than 90% of all cases [1,2]. The increasing global burden of diabetes is largely attributed to sedentary lifestyles, unbalanced diets, and aging populations, factors that collectively exacerbate the risk of cardiovascular, renal, ocular, and neurological complications [3].

Despite the diversity of pharmacological options—such as metformin, insulin analogs, DPP-4 inhibitors, GLP-1 receptor agonists, and SGLT2 inhibitors—achieving optimal glycemic control remains a significant challenge for many patients. Long-term use of these agents can lead to adverse effects, poor adherence, and increased financial burden, limiting their effectiveness in real-world settings [4]. Consequently, there is a growing scientific and clinical interest in natural compounds and complementary therapies that can provide safe, sustainable, and synergistic support to conventional pharmacotherapy.

Over the past decade, extensive research has focused on medicinal plants and bioactive phytochemicals with potential antidiabetic properties. Natural compounds have demonstrated antioxidant, anti-inflammatory, and insulin-sensitizing effects, regulating key molecular pathways involved in glucose metabolism, pancreatic β-cell protection, and glucose uptake [5,6,7].

Beyond phytochemicals, other natural interventions—such as those incorporating dietary fibers, probiotics, and functional foods such as apple cider vinegar—have shown promising results in improving glycemic variability, lipid metabolism, and gut microbiota composition [8,9]. The modulation of the gut–microbiota–pancreas axis appears particularly relevant, as it may enhance metabolic flexibility and reduce systemic inflammation, two hallmarks of T2DM pathogenesis.

Nevertheless, despite these encouraging findings, the safety profile and potential herb–drug interactions of natural compounds require careful assessment. To advance this field of research, future studies should prioritize integrative and multidisciplinary approaches, combining molecular, preclinical, and clinical data to elucidate mechanisms of action and optimize formulation strategies.

Collectively, the contributions presented in this Special Issue, “Natural Products in Diabetes Mellitus: 2nd Edition”, of *Pharmaceuticals* highlight the importance of evidence-based natural therapies as part of a broader, sustainable strategy for diabetes prevention and management.

## 2. Results

The articles in this Special Issue converge on the importance of natural compounds as adjuvants to antidiabetic therapy. The contributions are listed in the backmatter of this Editorial.

In the field of phenolic metabolites, Li et al. demonstrated that syringaldehyde attenuates hyperglycemia-induced cardiac hypertrophy via GLP-1R and AMPK. Similarly, Guerrero-Becerra et al. reviewed the role of polyphenols in Fabaceae, which have antioxidant and insulinotropic potential. Other emerging botanical resources include agarwood, evaluated in a scoping review (M. Fadzil et al.), which has shown antihyperglycemic and antioxidant effects, although it has not yet been included in clinical trials.

Considering specific antioxidants, Sandoval et al. showed that β-carotene supplementation protects pancreatic β-cells against ethanol-induced damage, using artificial intelligence as an innovative histological analysis tool. Huang et al. conducted a population-based study involving over 64,000 patients, finding that adjunctive Chinese herbal medicine reduced the risk of hearing loss in T2D patients. Likewise, Zhang et al. integrated evidence on polysaccharides, highlighting their ability to modulate gut microbiota and reduce diabetic complications.

The study conducted by Curiel Ayala et al. further expanded the diversity of approaches in this domain: chaya was critically reviewed as a source of flavonoids and dietary adjuvant, with warnings about interactions with metformin. Additionally, B. Abbas et al. suggested that a diosmin–linagliptin combination showed superior efficacy compared to monocompounds in rats, restoring hepatic metabolism and pancreatic function. Omoruyi et al. reported notably antioxidant, anti-inflammatory, and antidiabetic activities in *Kalanchoe pinnata*, and Karina et al. revealed the dual antidiabetic and antimicrobial potential of timbe (*Acaciella angustissima*). Rafailovska et al. indicated that cannabidiol (CBD) lowered glucose and improved hepatic and lipid parameters in animal models, and Cho et al. clearly showed that the combination of policosanol and banaba generated synergy in glycemic control, lipid profiling, and organ protection. Finally, Zeng et al. investigated how the Buzhong Yiqi Formula (BZYQF) controls postprandial glucose by inhibiting digestive enzymes, identifying flavonoids as the primary active compounds responsible.

The presented works coincide in their insight that natural compounds act through complementary mechanisms: incretin signaling (GLP-1R, AMPK), digestive enzymes (α-amylase, α-glucosidase), hepatic metabolic regulation, oxidative stress reduction, and gut microbiota modulation. A trend toward synergy is observed, both between botanical products and in combination with conventional drugs, as in the cases of diosmin–linagliptin and policosanol–banaba. Methodological innovations such as artificial intelligence, metabolomics, and multi-omics analysis are also emphasized.

## 3. Research Gaps and Perspectives

The integration of natural compounds into diabetes management faces several scientific and technological challenges, notably the scarcity of controlled clinical trials, lack of standardized phytochemical characterization, and limited assessment of herb–drug interactions and long-term safety [10,11]. Establishing harmonized extraction protocols and employing advanced analytical tools such as LC–MS, metabolomics, and fingerprint profiling are essential to ensure reproducibility and safety.

To enhance translational value, integrative multi-omics analyses are required to further elucidate host–microbiota interactions [12].

Recent advances in nanotechnology have also improved the bioavailability and stability of plant-derived compounds. Nanoencapsulation of curcuminoids, polyphenols, and berberine enhances their absorption and therapeutic efficacy, paving the way toward precision nutraceuticals [13,14].

Finally, from a sustainability perspective, the valorization of regional medicinal plants promotes circular bioeconomy principles and supports the UN Sustainable Development Goals—particularly SDGs 3 and 12 [2,15]. Integrating ethnobotanical knowledge with modern biomedical approaches will be key to developing safe, effective, and sustainable phytotherapeutics for diabetes and related disorders [5,6].

## 4. Conclusions

This Special Issue of *Pharmaceuticals*, “Natural Products in Diabetes Mellitus: 2nd Edition”, provides a broad overview of natural and complementary medicinal alternatives for diabetes management. The integrative evidence confirms the potential of phenolic compounds, polysaccharides, carotenoids, and herbal formulas, as well as the value of combined strategies. An immediate objective is progressing toward rigorous clinical trials that consolidate these options within contemporary therapeutics.
